# Cancer Immune Therapy for Philadelphia Chromosome-Negative Chronic Myeloproliferative Neoplasms

**DOI:** 10.3390/cancers12071763

**Published:** 2020-07-02

**Authors:** Morten Orebo Holmström, Hans Carl Hasselbalch, Mads Hald Andersen

**Affiliations:** 1 National Center for Cancer Immune Therapy, Department of Oncology, Herlev University Hospital, DK-2730 Herlev, Denmark; mads.hald.andersen@regionh.dk; 2 Department of Hematology, Zealand University Hospital, DK-4000 Roskilde, Denmark; hkhl@regionsjaelland.dk; 3 Department of Immunology and Microbiology, University of Copenhagen, DK-2200 Copenhagen N, Denmark

**Keywords:** myeloproliferative neoplasms, cancer immune therapy, JAK2, CALR, cancer vaccines, neo-antigens, immunoediting, immunosurveillance

## Abstract

Philadelphia chromosome-negative chronic myeloproliferative neoplasms (MPN) are neoplastic diseases of the hematopoietic stem cells in the bone marrow. MPN are characterized by chronic inflammation and immune dysregulation. Of interest, the potent immunostimulatory cytokine interferon-α has been used to treat MPN for decades. A deeper understanding of the anti-cancer immune response and of the different immune regulatory mechanisms in patients with MPN has paved the way for an increased perception of the potential of cancer immunotherapy in MPN. Therapeutic vaccination targeting the driver mutations in MPN is one recently described potential new treatment modality. Furthermore, T cells can directly react against regulatory immune cells because they recognize proteins like arginase and programmed death ligand 1 (PD-L1). Therapeutic vaccination with arginase or PD-L1 therefore offers a novel way to directly affect immune inhibitory pathways, potentially altering tolerance to tumor antigens like mutant CALR and mutant JAK2. Other therapeutic options that could be used in concert with therapeutic cancer vaccines are immune checkpoint–blocking antibodies and interferon-α. For more advanced MPN, adoptive cellular therapy is a potential option that needs more preclinical investigation. In this review, we summarize current knowledge about the immune system in MPN and discuss the many opportunities for anti-cancer immunotherapy in patients with MPN.

## 1. Introduction

In recent years, cancer immune therapy has received considerable attention, as this novel treatment modality can induce major responses and even cure patients with advanced cancer. In 2013, the academic journal *Science* designated cancer immune therapy as “breakthrough of the year”, and in 2018, James P Allison and Tasuko Honjo were awarded the Nobel Prize in Physiology or Medicine for their respective discovery of the canonical immunoregulatory systems cytotoxic T-lymphocyte antigen (CTLA)-4 and programmed death receptor (PD)-1/programmed death receptor ligand (PD-L)1, and cancer immune therapy is now used with great success in the treatment of several solid cancers.

In the setting of hematological malignancies, this therapy in the form of chimeric antigen receptor (CAR) T cells and immune-checkpoint-blocking antibodies has had the greatest impact in the treatment of lymphoblastic leukemia and lymphoma [1,2]. No clinical breakthroughs have been made, however, in the setting of myeloid leukemia, myelodysplastic syndromes and chronic myeloproliferative neoplasms (MPN). The last group consists of heterogeneous but closely related diseases of the hematopoietic stem cells (HSC) of the bone marrow. Several studies have shown that the immune system is deranged in MPN and that the reinstatement of competent tumor immune surveillance by cancer immune therapy could potentially be used to treat this group of chronic cancer diseases. In this article, we provide a short overview of MPN and the current treatment modalities. Next, we give an overview of the immune system in MPN and describe the evidence of the aforementioned immune deregulation in MPN. In addition, we offer some perspectives on potential cancer immunotherapeutic modalities that may prove successful in the setting of MPN.

### The Philadelphia Chromosome-Negative MPN

The Philadelphia chromosome-negative MPN comprise essential thrombocythemia (ET), polycythemia vera (PV) and primary myelofibrosis (PMF), and are all neoplastic diseases of the HSC. The symptoms and clinical findings in MPN patients overlap, and the diseases are sometimes difficult to distinguish from each other [3,4,5]. Patients with ET display an elevated amount of platelets in the peripheral blood, whereas patients with PV display an elevated red cell mass in the peripheral blood, usually in concert with leukocytosis and thrombocytosis. Both patients with ET and PV have an increased risk of thromboembolism and hemorrhage. In contrast to these *hyperproliferative* MPN is classical PMF—the advanced MPN disease, where patients display bone marrow fibrosis and cytopenia resulting in an increased risk of infections and hemorrhages. Patients with MPN have a significantly increased risk of acute myeloid leukemia (AML) [6]. The reported incidence of MPN varies from 1.15/100.000 to 4.99/100.000 [7], as does the reported prevalence: ET (11–42.851/100.000), PV (0.49–46.88/100.000) and PMF (1.76–4.05/100.000) [7]. Patients with MPN have a lower life expectancy than the background population [8] and have a lower quality of life compared to healthy controls [9,10]. However, patients with ET and PV may live for decades with their cancer disease [8].

Compared to other malignancies, the mutational landscape of MPN shows high homogeneity, as 98% of patients with PV and 50–60% of patients with PMF and ET harbor the Janus kinase 2 (*JAK2*) V617F mutation [11]. Approximately 70% of patients with wild-type (wt) *JAK2* ET and wt *JAK2* PMF have a mutation in exon 9 of the calreticulin (*CALR*) gene [12,13], and 10% harbor a mutation in the myeloproliferative leukemia protein (*MPL*) gene [14,15]. The *JAK2*V617-mutation induces a loss of function of the JH2 pseudokinase domain of JAK2, rendering JAK2 in a constitutively active state, resulting in the activation of downstream signaling pathways such as signal transducer and activator of transscription (STAT)3, STAT5, PI3K and MAPK/ERK, followed by increased cell proliferation [16]. Even though the oncogenic mechanism of the *CALR* mutations is not fully clarified, it appears that the mutations enhance the binding of CALR to the thrombopoietin receptor (TPO-R), resulting in the activation of TPO-R and ensuing megakaryocyte proliferation and thrombocytosis [17,18,19,20].

In most countries, the mainstay treatment for ET, PV and hyperproliferative PMF is cytoreductive therapy, such as hydroxyurea (HU) or anagrelide. The former is a weak chemotherapeutic agent, whereas the latter inhibits the maturation of platelets from hyperproliferating megakaryocytes. HU has been speculated to confer an increased risk of secondary malignancies, even though such a link has not yet been clearly established [21]. Another treatment option for hyperproliferative MPN is interferon-alpha (IFN-α), which can induce minimal residual disease and, in a subset of patients, normalization of the bone marrow [22]. In some patients, this normalization is sustained for years, even after the cessation of therapy [23]. The mechanisms of action of IFN-α have not yet been clearly established, but the drug is a potent immunostimulatory agent [24]. For several years, the treatment for hypoproliferative PMF has been supportive care (e.g., erythropoietin analogs, danazol and transfusions) but recently, the JAK1/2 inhibitor ruxolitinib has become available. As ruxolitinib is able to inhibit the activation of and thus signaling conferred by JAK1 and JAK2, the drug inhibits the aberrant JAK2-mediated signaling in MPN [25]. As the *CALR* and *MPL* mutations also rely on JAK2 to confer their oncogenic activation, ruxolitinib has an effect in patients with *JAK2*wt MPN too [26]. Ruxolitinib reduces splenomegaly and constitutional symptoms [27,28] and confers a modest survival benefit for patients [29]. Still, the only curative treatment modality for MPN is allogeneic hematopoietic stem cell transplantation [30], which is only used rarely due to the high treatment-related mortality.

## 2. The Immune System in MPN

### 2.1. Chronic Inflammation in MPN

Several studies have shown that the immune system is deregulated in MPN [31]. Patients with PV and ET have elevated levels of interleukin (IL)-6, IL-8, IL-12, tumor necrosis factor (TNF)-α and IFN-γ in the peripheral blood [32]. Tefferi et al. revealed deregulation in 20 of 30 analyzed cytokines in 127 PMF patients [33], and in another study, PMF patients displayed elevated levels of the inflammatory marker YKL-40 compared to healthy controls [34]. Most recently, a comprehensive cytokine profiling study has substantiated the significant role of elevated cytokines in disease phenotype and progression, providing a tool complementary to next generation sequencing in disease stratification and determination of the prognosis of patients with MPN [35]. Therefore, the marked effects of ruxolitinib on the constitutional symptoms in MPN are speculated to be conferred by the potent anti-inflammatory effects of this drug [36,37]. Several epidemiological studies have demonstrated a link between MPN and inflammatory/autoimmune diseases [38,39]. The evidence of cytokine derangements, chronic inflammation and an elevated risk of several inflammatory diseases in MPN suggests that MPN are an inflammatory model of human cancer development. In this model, chronic inflammation is hypothesized to induce the initial stem cell hit by inducing genomic instability and mutations, giving rise to MPN [40,41]. However, the theory of cancer-inducing inflammation is old and was already hypothesized by Virchow. Several lines of evidence show that chronic inflammation increases the risk of cancer at the inflamed site. As such, several chronic inflammatory and chronic infectious diseases have been linked to an increased risk of cancer [42]. This increased risk is believed to be mediated by mutagenic substances such as reactive oxygen species (ROS) and peroxynitrite that are produced by inflammatory cells at the inflamed site. After the initial stem cell hit, the transformed cells and tumor cells may co-exist and support the growth of one another by paracrine signaling in concert with the suppression of the tumor-specific immune response [43].

### 2.2. The Immune System in MPN

MPN are characterized by chronic inflammation, so both the cytokine environment and the immune system in general are deregulated [31]. Patients with PMF have fewer effector T-cells in the peripheral blood [44], and monocytosis is an independent adverse prognostic factor for overall survival in young patients with PMF [45] and in PV patients in general [46]. Riley et al. showed lower numbers of natural killer (NK) cells in the peripheral blood of MPN patients compared to healthy donors [47]. Regarding the number of regulatory T-cells (Treg) in MPN patients, data are conflicting, as two studies did not demonstrate any difference in peripheral blood Treg levels between MPN patients and healthy controls [48,49], whereas another study showed that patients with MPN have lower numbers of circulating Treg, and this difference was applicable for patients with ET, PV and PMF compared to healthy controls [50]. Two studies have investigated the number of myeloid-derived suppressor cells (MDSC) in the peripheral blood of MPN patients. MDSC are cells of myeloid origin that emerge in almost all cancer types and are able to suppress T-cell functionality through several mechanisms such as the release of the arginine-degrading enzyme arginase-I (ARG1), ROS and prostaglandins. A detailed description of the role of MDSC in cancer has been provided by Ostrand-Rosenberg and Gabrilovich [51,52]. Interestingly, MDSC from patients with MPN have a greater inhibitory potential against autologous CD3 + T cells compared to MDSC from healthy donors [53,54], and patient peripheral blood mononuclear cells (PBMC) show higher expression of ARG1 compared to healthy donor MDSC [53]. The *JAK2*V617F mutation is present in cells from the lymphoid compartment in some patients [55], and we recently identified *CALR* mutations in lymphoid cells from several patients with *CALR* exon 9 mutations [56]. Because of the important immune-related functions of both JAK2 and CALR [57,58,59,60,61,62], functional aspects of these mutations in the lymphoid compartment seem to be implicated, but the aspects of these mutations have yet to be elucidated.

### 2.3. Immune-Subversive Mechanisms in MPN

Apart from immune dysregulation in MPN, several other factors are believed to attenuate the tumor-specific immune response. Monocytes from PMF patients produce elevated amounts of the immunoregulatory cytokine transforming growth factor-beta (TGF-β) [63]. In addition, patients with PV have increased levels of terminally differentiated CD8+ T cells and lower levels of naïve T-cells compared to healthy age-matched controls. They also display lower levels of stable cell surface human leukocyte antigen (HLA)-I [64].

In gene expression profiling studies, Skov et al. showed a marked dysregulation of immune-related genes in MPN. Patients with MPN exhibit downregulation of human leucocyte antigen (HLA)-I, HLA-II, and HLA-related genes—such as beta-2-microglobulin, TAP1, TAP2 and CIITA [65]—and genes related to the activation and differentiation of lymphocytes were also reported to be downregulated [66]. Furthermore, MPN patients exhibit an upregulation of IL-4, which could lead to the enhanced expression of the anti-apoptotic protein survivin [67]. Patients with PV show upregulated IL-10, a cornerstone immunoregulatory cytokine, as well as a downregulation of CD40L and FAS, implying reduced antigen-presenting cell/T-cell interaction and decreased cytotoxic T-cell killing potential [67].

Romano and coworkers recently provided a detailed overview of the functionality of the immune system in MPN [68]. The main findings were that patients with PMF display lower levels of myeloid dendritic cells (moDCs) compared to healthy donors, and that moDCs from MPN patients display a lower priming potential compared to healthy-donor moDCs. Several important facts regarding immune cell functionality in *CALR*-mutant MPN were identified: moDCs in *CALR*-mutant PMF show decreased amounts of co-stimulatory molecules, *CALR*-mutant patients have a reduced Th1 compartment, and the Treg from patients with *CALR*-mutant PMF are more suppressive compared to Treg from *JAK2*-mutant patients and healthy donors [68]. As such, all of these findings could explain the tumor immune escape in MPN. In an elegant study, Prestipino et al. showed that *JAK2*V617F+ cells display increased levels of PD-L1 [69]. In a recent study, it was demonstrated that both CD4+ and CD8+ T cells, monocytes, and CD34+ hematopoietic stem cells from patients with MPN display an enhanced expression of both PD-L1 and PD-1 [70]. In addition to increased PD-1 expression, T cells from *CALR*-mutant patients also display increased expression of CTLA-4, and the in vitro treatment of patient T cells with PD-1- and CTLA-4-blocking antibodies enhances *CALR*-mutant-specific T-cell responses to CALR-mutant peptides [71]. Together, this suggests that T cells in patients with MPN are exhausted and thus not able to clear transformed cells, whereas the increased levels of PD-L1 on monocytes and HSC explains why T cells fail to kill the malignant cells.

Other potential but unproven immune escape mechanisms include platelet–T-cell interactions. Thrombocythemia confers a dismal prognosis in several types of solid malignancies [72]. The thrombocythemia commonly found in MPN patients has been speculated to give rise to increased cancer invasiveness and enhance the metastatic potential of second cancers through a “platelet–cancer loop” [73]. A likely explanation for this “adverse thrombocythemia” was recently providedas platelets from cancer patients bind to T cells and inhibit them through the release of TGF-β [74]. Of interest, MPN are “megakaryocytic neoplasms“ and usually exhibit thrombocythemia. As such, the thrombocythemia in patients could result in the higher binding of platelets to T cells in MPN, which, in turn, could impair T cell functionality through the binding of immunosuppressive TGF-β to TGF-β receptors on specific T cells.

### 2.4. Immunosuppressive Mechanisms Directly Mediated by JAK2V617F or CALR Exon 9 Mutations

The *JAK2*V617F mutation mediates its proliferative effects through STAT3 and STAT5 activation. Prestipino and coworkers showed that the JAK2V617F-mediated overactivation of STAT3 induces the enhanced expression of immunosuppressive PD-L1 on *JAK2*V617F-mutant cells [69]. Furthermore, the *JAK2*V617F mutation may modulate T-cell responses in another way by generating excessive ROS through the upregulation of Akt/PI3K, which in turn decreases levels of the ROS-converting enzyme catalase [75]. Because ROS potently inhibit T cell function [76,77], the excessive ROS generated by *JAK2*-mutant cells might dampen the JAK2V617F-specific immune response. CALR exon 9 mutations can also activate both JAK2 and PI3K/Akt [17], and *CALR*-mutant cells—similarly to *JAK2*V617F+ cells—may generate excessive amounts of ROS, thus suppressing T cell function. In addition, CALR is a chaperone important in the assembly of the HLA-I:peptide complex, and *CALR*-mutant cells display lower levels of HLA-I molecules [78]; however, this finding has been questioned by another study [71]. This hypothetical attenuated expression of HLA-I by *CALR*-mutant cells could pose an important immune escape mechanism, as the *CALR* mutations may potentially prevent the presentation of highly immunogenic neo-epitopes on mutant cells by the downregulation of HLA-I. Concurrently, CALR plays an important role in the generation of the cytolytic synapses of T cells [59,60,61,62], and *CALR* mutations have been identified in both T and B cells [56]. Thus, it is possible that cytotoxic T cells harboring a *CALR* mutation cannot exert its cytotoxic effector function.

**Figure 1:**
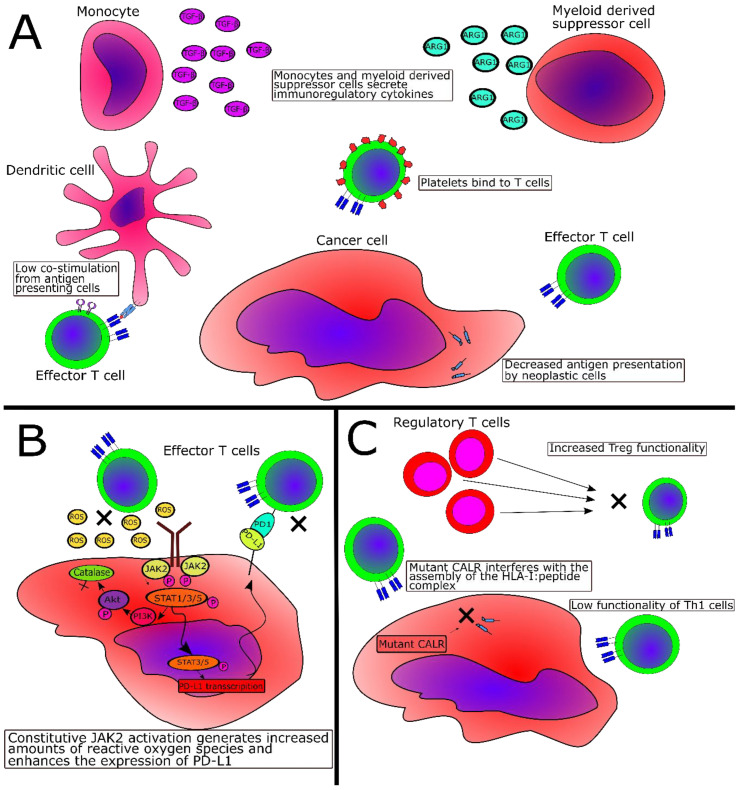
Suggested immune escape mechanisms in MPN. (**A**). Escape mechanisms that are shared between all MPN types, starting from the top and moving clockwise. Myeloid cells such as monocytes and myeloid-derived suppressor cells secrete TGF-β and ARG1, respectively. These proteins are highly immunosuppressive to T cells and thus induce immune suppression in the tumor microenvironment (TME). Platelets, which suppresses T cell reactivity, are released from the hyperproliferating megakaryocytes and bind to T cells, thereby suppressing tumor-specific T cells. Both *JAK2*- and *CALR*-mutant cells express lower levels of HLA-I molecules, resulting in decreased antigen presentation to T cells, thus facilitating tumor immune escape. Antigen-presenting cells express lower levels of costimulatory molecules, thus decreasing the priming of T cells. (**B**). Escape mechanisms that are employed, especially in *JAK2*-mutant MPN. The *JAK2*V617F mutation induces the activation of STAT1/3/5, whichh increases the activity of PI3K and Akt, thereby lowering the levels of the ROS-converting enzyme catalase. This results in increased levels of ROS in the TME, which decreases T cell reactivity. The *JAK2*V617F mutation induces the activation of STAT3/5, which enhances the transcripition of PD-L1, which is expressed on tumor cells, resulting in the anergy/exhaustion of activated PD-1-expressing tumor-specific T cells. (**C**). Escape mechanisms that are employed, especially in *CALR*-mutant MPN, starting from the top. Regulatory T-cells (Treg) from patients with *CALR-*mutant MPN are more suppressive than Treg from healthy donors. As the calreticulin protein is an important component in the assembly of the HLA-I:peptide complex, the *CALR* mutations were speculated to attenuate the expression of antigens on HLA-I molecules, which has now been shown [78]. Effector T-cells from patients with *CALR*-mutant MPN are less functional than effector T-cells from healthy donors.

Patients with PMF display increased levels of freely circulating CALR compared to healthy controls, but no difference in CALR levels was identified between patients with *JAK2*V617F+ and *CALR*-mutant PMF [79]. Additionally, it has been demonstrated that mutant CALR protein is not retained within the mutant cell but released into the extracellular space [80]. Garbati and coworkers showed that the stimulation of monocytes with supernatants from *CALR*-mutant cells enhanced the expression of IL-10 and other immunosuppressive cytokines. The authors proposed that full-length mutant CALR released from mutant cells into the supernatant is the factor responsible for the enhanced release of monocyte-derived immunosuppressive factors [81]. In concert with these findings are recently published data showing that *CALR* mutations may abrogate the effect of immunogenic cell death (ICD) [82]. ICD is a process where dying cells express prophagocytic surface molecules, one of the most important being CALR, thereby enhancing the DC-mediated phagocytosis of the cells, which allows for the more effective priming of T cells specific for antigens in the dying cell [83]. As such, it could be speculated that *CALR*-mutant cells release mutant CALR protein that not only induces immunosuppression through the stimulation of monocytes but also prevents the effective phagocytosis of malignant cells, hence preventing the proper priming of T cells specific for CALR-mutant epitopes, which in total would negate the effect of immunogenic cell death. Figure 1 summarizes some of the proposed immune escape mechanisms in MPN, and Table 1 provides a schematic overview of these.

### 2.5. Evidence of Tumor Immne Escape in MPN

**Figure 2:**
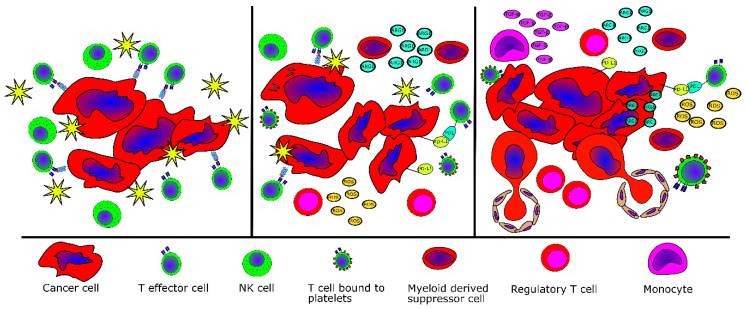
The disease continuum in MPN related to tumor elimination, equilibrium and escape. Left panel. The neoplastic cells are cleared by effector immune cells—T cells and NK cells. Middle panel. The immune system cannot clear neoplastic cells completely because immunosuppressive cells have now entered the tumor microenvironment and the cancer cells themselves employ immunosuppressive mechanisms. In MPN, this disease stage would be essential thrombocythemia (ET), polycythemia vera (PV) or prefibrotic/early myelofibrosis. Right panel. The immune system cannot prevent the evolution and metastasis of the malignant cells because the bone marrow is flooded with immunosuppressive cytokines such as IL-10 and ARG1 in addition to ROS released from *JAK2*-mutant cells. In MPN, this disease stage would be advanced myelofibrosis.

These findings suggest that the immune system is dysregulated in MPN and that MPN develops and evolves because of tumor immune evasion. Supporting this notion is the fact that even though *CALR* mutations are highly immunogenic [84] and patient T cells are highly reactive to the mutations [85], *CALR*-mutant cells in patients escape immune-mediated elimination. Interestingly, compared to patients with early MPN (ET), patients with advanced MPN (PMF) have fewer and weaker T-cell responses to mutant CALR epitopes [84]. This finding fits into the general concept of tumor-specific immune responses, which stipulates that the immune system in patients with advanced cancer is exhausted and hyporeactive [86]. As the *CALR* mutations are cancer-specific mutations, healthy donor T cells are not expected to display any reactivity against mutant CALR epitopes. However, most surprisingly, we have shown that healthy donors harbor T cells specific to mutant CALR epitopes. These responses are stronger and more frequent than in patients with *CALR*-mutant MPN, and healthy donor T cells specific to the mutant CALR epitopes are memory T-cells [87]. This implies that specific T cells in healthy donors are antigen experienced, i.e., they have been challenged with mutant CALR epitopes previously. An explanation for this could be that healthy individuals allegedly acquire a *CALR* exon 9 mutation, but given the high immunogenicity of the mutations, the immune system clears all the mutant cells, and the generation of T-cell memory specific to the mutant CALR ensues. The isolation, using tetramers, of CALR-mutant-specific T cells from healthy donor PBMC offers further evidence for this concept [88]. The high frequency of CALR-mutant-specific immune responses in healthy donors suggests that only a very low proportion of healthy donors harbor CALR exon 9 mutations. This was recently proved in a Danish population-based study where 19,958 individuals were analyzed for the occurrence of the *JAK2*V617F and *CALR* exon 9 mutations. Interestingly, 3.1% of the total population were *JAK2*V617F+, whereas only 0.16% harbored a *CALR* mutation, giving a ratio of *JAK2*V617F to *CALR*-mutation of 19:1 [89]. By contrast, the ratio is only 5.6:1 in patients [89]. This suggests that healthy donors clear the *CALR*-mutant cells before they establish and expand in the bone marrow. After a median follow up of 6.2 years, four of the *CALR*-mutant healthy donors identified in the above-mentioned study underwent full medical examination including bone marrow biopsy to clarify if they had developed overt *CALR*-mutant MPN [90]. Interestingly, none of the healthy donors had developed MPN, and all the donors displayed strong T-cell responses to several CALR-mutant epitopes [90]. Taken together, these data support the hypothesis of cancer immunoediting [86,91] in MPN: healthy donors display T-cell memory to mutant CALR epitopes (elimination) [87], healthy donors with CALR-mutant cells in the peripheral blood show strong T-cell responses to CALR mutations (editing) [90], and patients with overt *CALR*-mutant MPN show weaker and less frequent CALR-mutant-specific immune responses compared to healthy donors (escape) [84] (Figure 2). Giving impetus to the notion of tumor immune escape in MPN, it has been demonstrated that patients with MPN have an increased risk of several cancers both before and after the diagnosis of MPN [92,93,94].

## 3. Potential Cancer Immune Therapeutic Strategies for MPN

### 3.1. The Role of IFN-α in MPN

In light of the above, the reinstatement of effective tumor immune surveillance seems to be an intriguing treatment option for MPN. IFN-α has been used to treat MPN and other malignancies for decades [95], and the mechanism of action is speculated to be its potent immunostimulatory effects [24]. IFN-α enhances antigen presentation, cross-priming, the maturation of dendritic cells, and the cytotoxic capabilities of cytotoxic T-lymphocytes and NK cells and suppresses the inhibitory capacity of MDSC and Treg. Data from murine studies indicate that type I interferons are responsible for the effects of anthracyclines, cyclophosphamide, radiotherapy, immune checkpoint inhibitors and allogeneic hematopoietic stem cell transplantation [95,96,97,98,99]. As such, the treatment of MPN with IFN-α probably partially reinstates otherwise-defective tumor immune surveillance, as IFN-α can induce a complete hematological response and significantly reduce both the *JAK2*-mutated [100] and *CALR*-mutated allele burden [101,102]. Moreover, a subset of patients experience normalization of the bone marrow and prolonged hematological responses for years, even after cessation of IFN-α therapy [23]. This effect suggests that IFN-α enhances the immune response against the malignant cells. Riley et al. demonstrated that treatment with IFN-α resulted in marked alterations in the immune phenotype of patients with MPN, further supporting this notion [47,49,103]. Additionally, treatment with IFN-α enhances the expression of both class I and class II HLA genes, implying that IFN-α not only potentiates immune effector cells but also sensitizes the target cells to immune-mediated destruction [104]. Another study on circulating Treg in IFN-α-treated patients showed an increase in circulating Treg after therapy [105]. Taken together, these studies suggest that treatment with IFN-α might generate an effective anti-tumor immune response by inducing alterations in the immune phenotype and enhancing antigen presentation by malignant cells.

### 3.2. Recognition of Neoplastic Cells Through Antigen Recognition by T cells

Cancer immune therapy exploits the immune-mediated recognition of malignant cells, and this recognition depends mainly on the recognition of tumor antigens by T cells. There are several types of tumor antigens that may be recognized by T cells [106]. Generally, there are two classes of antigens that may be targeted by the immune system—self antigens and non-self antigens. One class of self antigens are so-called overexpressed antigens, which are expressed by healthy cells but overexpressed by neoplastic cells (e.g., HER2/Neu in several epithelial tumors and WT1 in AML). Another class of self antigens are differentiation antigens, which are only expressed by a very limited class of cells and by tumor cells (e.g., melanoma antigen recognized by T-cells (MART) that isexpressed by melanocytes and in melanoma). The cancer germline antigens (CGA), which are also called cancer testes antigens, are another type of self antigens that are expressed only by cancer cells or by cells in immunoprivileged tissues. The CGA are believed to be more immunogenic than the two other types of self antigens, as high-affinity CGA-specific T-cells have a lower chance of being deleted during the development of central tolerance [106]. Non-self antigens include viral antigens, which are highly immunognenic due to their non-human origin. The last type of non-self antigens are the so-called neo-antigens, which arise from acquired somatic mutations in cancer cells. These antigens are of great interest as targets for cancer immune therapy and are belived to have superior immunogenic potential compared to the other types of antigens. This is explained by the fact that neo-antigen-specific T-cells are not deleted during the development of central tolerance [107], and enhancing the neo-antigen-specific immune response through cancer immune therapy such as therapeutic cancer vaccination is an intriguing treatment option.

### 3.3. Cancer Immune Therapy for MPN—Targeting the Neoantigens

However, one inherent problem with targeting specific neoantigens through, for example, therapeutic cancer vaccines is that somatic mutations vary both between patients and between different cancers, thereby resulting in a vast plethora of different neo-antigens that are shared neither between patients nor diseases. As such, one would need to develop personalized cancer vaccines through the sequencing of the mutatonome of each patient—a feat that is possible but comes at a high cost. However, in MPN, there is no such need to target personal neo-antigens as 80–90% of patients harbor either the *JAK2*V617F or *CALR* mutations. Additionally, we recently reported that the immune system can selectively target cells carrying the *JAK2*V617F and *CALR* exon 9 mutations [84,85,108]. As noted above, it seems likely that patients with *CALR*-mutant MPN have developed disease because of tumor immune escape. The reinstatement of a competent anti-tumor immune response by therapeutic cancer vaccination might result in tumor cell clearance. As such, therapeutic cancer vaccination targeting the driver mutations in MPN may be a potential new treatment modality. For this reason, we have finalized a phase I clinical vaccination trial with a CALR-mutant epitope for patients with *CALR*-mutant MPN (NCT03566446), and clinical, molecular and immunological analyses are ongoing. Just recently, using in silico prediction analyses, Schischlik and colleagues showed that both the *CALR* and *MPL* mutations generate neo-antigens that may bind to common HLA-I molecules, and additionally showed that the *SF3B1* mutations that are identified occasionally in MPN may be a rich source of neo-antigens that may be targeted by cancer immune therapy [109]. In this conjuncture, it would be worthwhile to investigate the frequency of certain HLA types in patients with *CALR*-mutant MPN, as it has been demonstrated that certain HLA types are underrepresented in cancers with immunogenic driver mutations. Patients with HLA-A3 and HLA-B8 and, especially, patients co-expressing these HLA types are underrepresented in patients with *BCR-ABL*+ chronic myeloid leukemia [110], and HLA-B*07, B*18 and B*40 are underrepresented in patients with nucleophosmine-1 mutant AML [111]. As both of these mutations generate immunogenic neo-antigens [112,113], it is speculated that patients with the above-mentioned HLA types process and present highly immunogenic epitopes on transformed cells, thus facilitating immune-mediated tumor rejection.

### 3.4. Targeting of Immunoregulatory Mechanisms in MPN

Therapeutic cancer vaccination against the *JAK2*V617F and *CALR* exon 9 driver mutations is likely to induce immune responses. However, the immune dysregulation in MPN could potentially prevent tumor-specific immune responses and negate any clinical effect of vaccines. As such, targeting regulatory mechanisms in the tumor microenvironment may also be important. PD-1 is a key regulatory surface molecule that delivers inhibitory signals to maintain T cells’ functional silence against their cognate antigens [114]. The discovery of how PD-1 on T cells binds to PD-L1 on tumor cells or other cells in the tumor microenvironment has increased our understanding of how tumors that are flooded with tumor-infiltrating lymphocytes evade immune-mediated destruction [115]. PD-1 is expressed by activated T cells, and the binding of PD-1 to PD-L1+ cells in the tumor microenvironment renders the T cell anergic. This phenomenon has been exploited clinically by the invention of PD-1-blocking antibodies, which block the PD-1/PD-L1 interaction. Treatment with these immune-checkpoint-blocking antibodies has shown outstanding results in a variety of solid tumors, [116,117] and naturally, the PD-1-blocking antibodies have been tested in hematological cancers. The compound has shown remarkable clinical effects in the treatment of relapsed/refractory classical Hodgkin lymphoma [118]. Results from clinical trials with other hematological cancers have been less promising, albeit some studies suggest that PD-1 blockade may be more efficacious in cancers with high PD-L1 expression. As such, patients with activated B-cell (ABC)-subtype diffuse large B cell lymphoma (DLBCL) obtain more benefit from PD-1 blockade than patients with germinal center B-cell (GCB)-subtye DLBCL, which could be due to the increased expression of PD-L1 in ABC-subtype DLBCL [119]. Hence, an increased expression of PD-L1 by tumor cells could be a predictive biomarker of PD-1 blockade sensitivity. However, clinical trials with PD-1 blockers in patients with multiple myeloma (MM) have partially contradicted this notion. Myeloma cells express increased levels of PD-L1 [120], and as such, PD-1/PD-L1-targeting therapy should be ideal for patients with MM, but PD-1 blockade has not displayed any relevant clinical effect in MM [119]. PD-1-blocking antibodies have been tested in myelodysplastic syndrome (MDS) and AML. The rationale for this is the fact that patient peripheral blood T cells express higher levels of PD-1 at relapse [121], and treatment with with clinically approved hypomethylating agents such as 5-azacytidine and 5-aza-2′deoxycitidine increases the expression of PD-L1 on transformed cells and PD-1 on T cells [122,123]. PD-1-blocking antibodies, especially in combination with hypomethylating agents, have shown promising results in relapsed/refractory AML, with an overall response rate of 33% [124]. Taking the above into account, the linical testing of PD-1-blocking antibodies in MPN seems highly relevant given the fact that the *JAK2*V617F mutation enhances the expression of PD-L1 on transformed cells [69]. Taking the increased risk of autoimmune disease in patients with MPN into account, one needs to carefully consider the use of PD-1-blocking antibodies, as treatment with these frequently results in the emergence of autoimmune phenomena [125]. To date, only one study is evaluating the clinical efficacy of immune checkpoint inhibitors in patients with MPN (NCT03065400), but no clinical results have been reported at the time of the writing of this paper. T cells from one patient included in the above-mentioned trial showed enhanced reactivity to *CALR*-mutant epitopes after treatment in vivo and in vitro with PD-1-blocking antibodies [71], thus supporting the idea of enhancing T-cell responses in patients with MPN by immune checkpoint inhibitors.

**Figure 3:**
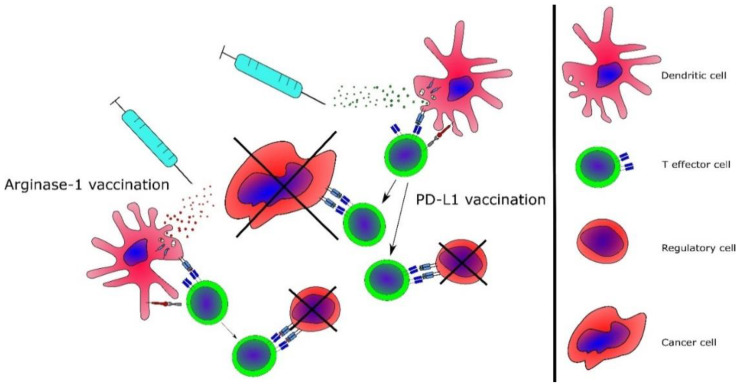
Therapeutic cancer vaccination targeting immunosuppressive mechanisms in MPN. Left. Vaccination with arginase-1 (ARG1)-derived epitopes will enhance the ARG1-specific T-cell response, leading to the recognition and killing of ARG1-producing cells and resulting in decreased amounts of ARG1 in the tumor microenvironment. Right. Vaccination with PD-L1-derived epitopes will induce a T-cell response against regulatory cells and against *JAK2*V617F-mutant cells.

Interestingly, the immune system itself can target regulatory cells, and naturally occurring T cells specific to epitopes from pivotal immune regulatory proteins such as indoleamine 2,3-dioxygenase, PD-L1, PD-L2 and ARG1 have been described in cancer patients [126,127,128,129]. The PD-L1-peptide co-stimulation of T-cell cultures in vitro increases the immune response against viral antigens [130] and enhances the immunogenicity of a dendritic cell-based cancer vaccine [131]. As such, the vaccination of cancer patients with epitopes from these immunoregulatory proteins is likely to induce the formation of anti-regulatory T cells, which would enhance the tumor-specific immune response [132]. As noted above, patients with MPN have increased levels of the immunoregulatory proteins ARG1 and PD-L1 [53,69]. Of interest, patients with MPN have frequent and strong T-cell responses against both of these regulatory proteins [133,134], and vaccination with these epitopes could potentially target immunoregulatory mechanisms and neoplastic cells in MPN (Figure 3). Patients with non-advanced MPN (e.g., ET) have stronger and more frequent responses compared to patients with advanced MPN (e.g., PMF) [133,134]. These data are in line with the notion that patients with non-advanced cancer have a more potent tumor-specific immune response [86]. This pattern has also been demonstrated clinically: patients with non-advanced cancer have the best clinical responses to therapy [135,136]. In the setting of MPN, the implication is that patients with ET and PV are more likely to have a response to therapeutic cancer vaccination. By contrast, patients with advanced PMF should be considered for other, more advanced treatment modalities. We believe that the immune responses elicited by vaccinations with the JAK2-mutated or CALR-mutated antigens may be enhanced by vaccination against one or more immunoregulatory mechanisms, such as PD-L1 and ARG1. As the expression of the cornerstone immunoregulatory proteins PD-L1 and ARG1 is increased in MPN, we speculate that vaccination against both of these will enhance the anti-tumor immune response in patients, and we have consequently launched a phase I/II clinical vaccination trial with PD-L1- and ARG1-derived epitopes in patients with MPN (NCT04051307).

### 3.5. Advanced Cancer Immune Therapeutic Strategies for MPN

For patients with advanced myelofibrosis, peptide vaccination is not believed to induce an effective anti-tumor immune response, and patients with advanced PMF exhibit both functional and quantitative immune defects, as described above. As such, another advanced, yet feasible, treatment modality for these patients would be adoptive T-cell therapy, in which tumor-reactive autologous T cells are infused into the patient. These tumor-reactive T cells can be grown from tumor-infiltrating lymphocytes (TIL) [137]. Obtaining TIL from patients with PMF would require obtaining bone marrow cells, which is not possible in these patients because of the fibrotic marrow (i.e., “dry tap”). However, it is possible to expand a T-cell culture specific for a given tumor-specific antigen (e.g., JAK2V617F), clone the JAK2V617F-specific T-cell receptor (TCR) and transduce the TCR into autologous T cells. These cells could then be expanded and reinfused into the patient [137]. The development of such treatment modalities is in the early stages, but the possibilities are intriguing because patients with advanced MPN could possibly reap great benefits from this treatment. However, the potential of off-target effects of the TCR of interest requires close assessment. Especially in the setting of the *JAK2*V617F mutation, which is a single amino acid substitution, cross-reactivity with the wt epitope is a risk and could result in deleterious side effects.

**Figure 4:**
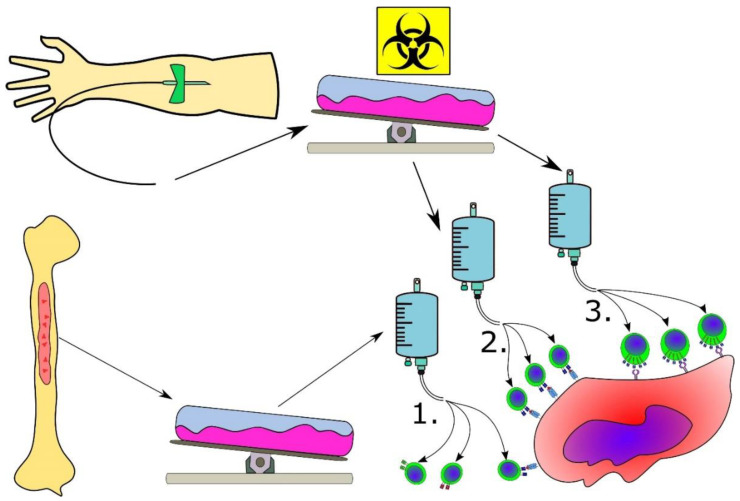
Cytotherapeutic modalities for MPN. (**1**). Tumor-infiltrating lymphocytes (TIL) are isolated from the bone marrow of the patient, expanded and reinfused into the patient. Only some of the TIL are specific to the neoplastic cells in the bone marrow. These are displayed by the T cell with the blue T-cell receptor (TCR), which is the only TCR specific for the antigen of interest. (**2**). Peripheral blood mononuclear cells (PBMCs) are isolated from the peripheral blood of the patient, and the PBMCs are transduced or transfected in bioreactors to express a TCR specific for an antigen expressed by the cancer cell. This is displayed by all the T cells that have a TCR specific for the antigen of interest (blue TCR). (**3**). PBMC are isolated from the peripheral blood of the patient and are transduced with DNA that encodes a chimeric antigen receptor (CAR). This CAR combines the promiscuity of the B cell receptor (no need for presentation on HLA molecules) with the power of the TCR, allowing the CAR T-cell to be activated and initiate cytotoxic killing upon binding to a surface molecule on the target cells. In the setting of MPN, this antigen expressed on the surface of the cell could be mutant CALR.

The treatment of patients with acute lymphoblastic leukemia and non-Hodgkin lymphoma with CART and bispecific T-cell engagers (BiTE) has shown remarkable results, with overwhelming response rates in heavily pre-treated patients [1,138]. These modalities target CD19, which is a surface molecule commonly expressed in several B-cell malignancies, and treatment with CD19-specific CART results in chronic B-cell aplasia in a substantial proportion of patients due to the cytotoxic killing of both tumor cells and B-cell precursors. Although this B-cell aplasia is problematic, patients can live with this condition. However, the treatment of MPN with CART and BiTE is challenging because no surface antigen has been shown to be genuinely expressed by the neoplastic cells. Targeting hematopoietic stem cell markers expressed by malignant cells in MPN, such as CD33 and CD34, would likely result in the killing of both neoplastic and normal hematopoietic stem cells and is thus precluded. Mutant CALR is expressed on the plasma membrane and binds the thrombopoietin receptor [17]. If this binding of mutant CALR to the thrombopoietin receptor is stable, the possibility of CALR-mutant-specific monoclonal antibodies might be worth exploring. An illustration of the potential different cytotherapeutic modalities for MPN is provided in Figure 4.

### 3.6. Combinatorial Strategies Involving Cancer Immune Therapy

Cancer vaccines represent a promising means of eliminating minimal residual disease without inducing toxicity and secondary malignancies [139]. However, to date they have largely failed to demonstrate a significant improvement of patient outcomes, probably reflecting the ability of malignant cells to suppress the function of the induced immune cells. As suggested above, co-vaccination against ARG1 and/or PD-L1 may be synergistic. Another exciting approach is combining it with checkpoint-blocking antibodies directed against the PD-1/PD-L1 pathway. In practice, antibodies that target the inhibitory checkpoints elicit impressive, dynamic and durable tumor regression and boost anti-cancer immune responses. Given the biological effects of IFN-α, the combination of IFN-α with vaccination seems logical. Vaccination would prime the immune system to target cells carrying the mutated antigen, and IFN-α would further enhance the induced anti-cancer immune response to eliminate malignant cells. Another intriguing treatment modality would be to exploit ICD, a form of regulated cell death that can activate the adaptive immune response and induce an immune response against antigens from the dead cells [83,140]. Certain chemotherapeutic agents and radiation induce ICD, and their clinical effects are speculated to depend mainly on ICD. This association may explain the abscopal influence of radiation therapy in which tumor masses *outside* the radiation field shrink after radiation therapy [83]. One of the hallmarks of ICD is the emergence of CALR on the plasma membrane. Obeid et al. showed that radiation or treatment with anthracyclines induces the surface exposure of CALR and that this exposure leads to the enhanced phagocytosis of tumor cells by dendritic cells. Furthermore, CALR-mediated phagocytosis enhances IFN-γ release in tumor-draining lymph nodes, and radiation and anthracycline therapies appear to depend on an intact T-cell response because T-cell–depleted mice do not respond to treatment [141,142]. However, the study by Liu et al. mentioned above poses the idea that the induction of ICD in patients with *CALR*-mutant MPN will not induce a tumor-specific immune response [82]. Hence, the freely circulating mutant CALR produced by transformed cells will be phagocytosed by APCs and, consequently, abrogate thephagocytosis of transformed cells by the APC. However, the induction of ICD in patients with *JAK2*V617F^+^ MPN might still seem as an option worth investigating, especially as the *JAK2*V617F-mutation is not nearly as immunogenic as the *CALR* exon 9 mutations. As such, an enhanced tumor specific immune response to *JAK2*V617F^+^ cells through ICD might be what is needed to induce a *JAK2*V617F^+^-specific immune response.

## 4. Conclusions

MPN are associated with a deregulation of the immune system, and cancer immune escape is likely an important factor in the development and evolution of the disease. However, this immune dysregulation could be reverted by several therapeutic options to reinstate tumor immune surveillance. Vaccination with JAK2-mutant- and CALR-mutant-derived peptides will probably induce tumor-specific immune responses that will be further enhanced by co-vaccination with anti-regulatory epitopes, such as PD-L1- and ARG1-derived epitopes. Other therapeutic options that could be used with therapeutic cancer vaccines are immune checkpoint–blocking antibodies and IFN-α. For patients with more advanced MPN, adoptive T-cell therapy with TIL or TCR-transduced T cells is a potential option but needs more preclinical investigation. The lack of proper extracellular targets for CART and BiTEs make these two otherwise-intriguing treatment modalities unlikely candidates.

## Figures and Tables

**Table 1:** Immune-suppressive mechanisms identified in myeloproliferative neoplasms (MPN). Primary myelofibrosis (PMF), natural killer cells (NK cells), myeloid derived suppressor cells (MDSC), human leucocyte antigen (HLA), polycythemia vera (PV).

Escape Mechanism	MPN Type	Reference
Fewer effector T cells in peripheral blood	PMF	[40]
Fewer NK cells in peripheral blood	All MPN	[43]
Elevated numbers of MDSC in peripheral blood	All MPN	[47,48]
Higher inhibitory potential of MDSC in peripheral blood	All MPN	[47]
Elevated TGFβ production by bone-marrow-derived monocytes	PMF	[57]
Lower levels of naïve T-cells and higher levels of terminally differentiated effector T-cells	PV	[58]
Lower levels HLA-I on monocytes	PV	[58]
Lower levels of HLA-I on mutant cells	*CALR*-mutant MPN	[72]
Deregulation of genes related to antigen processing and activation as well as immune cell activation and inflammation	All MPN	[59,60,61]
Lower number of myeloid dendritic cells and lower priming potential of myeloid dendritic cells	PMF (lower number of myeloid dendritic cells), all MPN	[62]
Reduced Th1 compartment and more suppressive regulatory T-cells	*CALR*-mutant MPN	[62]
Increased expression of PD-L1 on *JAK2*V617F^+^ cells	*JAK2*V617F^+^ MPN	[63]
Increased expression of PD-1 and PD-L1 on CD4+ and CD8^+^ T-cells, monocytes and CD34^+^ hematopoietic stem cells	All MPN	[64]
Increased expression of PD-1 and CTLA-4 on T cells	All MPN	[65]
Overproduction of reactive oxygen species	*JAK2*V617F^+^ MPN	[69]
Overproduction of IL-10 and additional immunosuppressive cytokines by monocytes	*CALR*-mutant MPN	[75]
Abrogation of immunogenic cell death	*CALR*-mutant MPN	[76]
